# Effects of hypertonic saline and mannitol on cortical cerebral microcirculation in a rabbit craniotomy model

**DOI:** 10.1186/s12871-015-0067-z

**Published:** 2015-06-09

**Authors:** Pavel Dostal, Jitka Schreiberova, Vlasta Dostalova, Vlasta Dostalova, Tomas Tyll, Jiri Paral, Islam Abdo, Miroslav Cihlo, David Astapenko, Zdenek Turek

**Affiliations:** 1 Department of Anesthesia and Intensive Care Medicine, Charles University, Faculty of Medicine Hradec Kralove, University Hospital Hradec Kralove, Hradec Kralove, Czech Republic; 2 Department of Anesthesia and Intensive Care Medicine, Charles University, 1st Faculty of Medicine Prague, Military University Hospital Prague, Prague, Czech Republic; 3 Department of Neurosurgery, Charles University, Faculty of Medicine Hradec Kralove, University Hospital Hradec Kralove, Hradec Kralove, Czech Republic; 4 Department of Military Surgery, Faculty of Military Health Sciences, Hradec Kralove, University of Defence, Brno, Czech Republic

**Keywords:** Brain, Hypertonic saline, Mannitol, Microcirculation, Osmotherapy

## Abstract

**Background:**

Hyperosmolar solutions have been used in neurosurgery to modify brain bulk and prevent neurological deterioration. The aim of this animal study was to compare the short-term effects of equivolemic, equiosmolar solutions of mannitol and hypertonic saline (HTS) on cerebral cortical microcirculation in a rabbit craniotomy model.

**Methods:**

Rabbits (weight, 2.0–3.0 kg) were anesthetized, ventilated mechanically, and subjected to a craniotomy. The animals were allocated randomly to receive a 3.75 ml/kg intravenous infusion of either 3.2 % HTS (group HTS, n = 8) or 20 % mannitol (group MTL, n = 8). Microcirculation in the cerebral cortex was evaluated using sidestream dark-field (SDF) imaging before and 20 min after the end of the 15-min HTS infusion. Global hemodynamic data were recorded, and blood samples for laboratory analysis were obtained at the time of SDF image recording.

**Results:**

No differences in the microcirculatory parameters were observed between the groups before the use of osmotherapy. After osmotherapy, lower proportions of perfused small vessel density (P = 0.0474), perfused vessel density (P = 0.0457), and microvascular flow index (P = 0.0207) were observed in the MTL group compared with those in the HTS group.

**Conclusions:**

Our findings suggest that an equivolemic, equiosmolar HTS solution better preserves perfusion of cortical brain microcirculation compared to MTL in a rabbit craniotomy model.

## Background

Hyperosmolar solutions have been used during neurosurgical procedures to improve operating conditions and prevent transdural herniation and neurological deterioration [1–3]. Mannitol (MTL) and hypertonic saline (HTS) have been evaluated in patients without intracranial hypertension [1, 2, 4, 5] with equivocal results. A recent meta-analysis comparing the intraoperative effects of HTS and MTL during neurosurgical procedures [6] demonstrated significantly increased odds of satisfactory intraoperative brain relaxation and intracranial pressure control using the former agent.

Administering HTS or MTL increases serum osmolarity and decreases intracranial pressure and brain water content in non-injured brain areas, as shown in animal studies [7, 8]. The effectiveness of a hyperosmolar solute depends on its permeability through an intact blood–brain barrier (BBB). The BBB is almost impermeable to HTS (reflection coefficient [RC] = 1) but more permeable to MTL (RC = 0.9) [5, 9]. Therefore, HTS may induce a more intensive fluid shift from brain tissues into the intravascular space compared with that of MTL.

Other biochemical and physiological differences exist between HTS and MTL. HTS acts to increase the effective circulating volume, whereas MTL decreases the circulating volume through diuresis [6, 10, 11]. HTS results in improved cardiac output, improved regional blood flow, improved cerebrospinal fluid absorption [10], and beneficial immunomodulation [12]. HTS has also been suggested to be superior to MTL for brain oxygenation and cerebral hemodynamics [13]. However, MTL may improve microvascular cerebral blood flow [14], reduce blood viscosity [15], improve blood rheology [16], reduce cerebrospinal fluid production [17], promote free radical scavenging [18], and inhibit apoptosis [19].

Alterations in microcirculation can be investigated using sidestream dark-field (SDF) imaging (MicroScan; MicroVision Medical, Amsterdam, The Netherlands). SDF imaging is a well-validated, stroboscopic LED ring-based imaging modality introduced to observe microcirculation clinically [20]. SDF technology has been used to study changes in the microcirculation under various clinical conditions in both animal [21] and human studies [22]. SDF has also been used to investigate cortical brain microcirculation in animals with different pathological conditions, mainly in models of sepsis [23–26].

The aim of this animal study was to compare the effects of equivolemic, equiosmolar solutions of MTL and HTS on cerebral cortical microcirculation in a rabbit craniotomy model.

## Methods

### Animals

All experimental procedures were performed after approval from the Animal Welfare Body of the Charles University in Prague, Faculty of Medicine in Hradec Kralove, Czech Republic (approval no. 17-16/2014-6848) in accordance with Czech legislation on the protection of animals, which complies with Directive 2010/63/EU of the European Parliament and Council. Ten male and female rabbits (New Zealand white rabbit; weight, 2.0–3.0 kg; VELAZ 34081/2008-10001, CZ 21906828, Únětice, Czech Republic) were included in the study. The animals were housed in a standard cage at 21 °C under a 12-h dark/12-h light cycle with unrestricted access to laboratory chow and tap water. After a 1-week acclimatization period, the rabbits were used for the study.

### Anesthesia and surgical preparation

After an overnight fast with unrestricted access to tap water, the rabbits were anesthetized using an intramuscular induction dose of ketamine (40 mg/kg) and xylazine (4 mg/kg). The animals were placed in the supine position on an operating table. The body locations used for cannulation, electrocardiogram electrodes, and tracheostomy were shaved. Intravascular cannulas (Vasofix® Safety, B.Braun, Melsungen, Germany) were inserted in both marginal ear veins (G24) and the right central ear artery (G22) for continuous blood pressure monitoring, arterial blood gas analysis, and continuous infusion of a balanced crystaloid solution (Ringerfundin, B. Braun, Melsungen, Germany, 10 ml/kg/h), anesthesia, and a muscle relaxant. Mean arterial blood pressure was maintained above 50 mmHg with norepinephrine infusion as necessary.

The animals were tracheotomized after they were stabilized hemodynamically. A cuffless tracheal tube with an outer diameter of 2.5 mm was inserted between the third and fourth tracheal rings. After verifying correct placement by auscultation, mechanical ventilation was initiated using an anesthesia machine (Cirrus Trans2/Vent 2, Datex, Helsinki, Finland) with initial settings of pressure-controlled ventilation, respiratory rate of 40 breaths/min, inspiratory pressure of 14–16 cm H_2_O according to the weight of the rabbit, and a positive end-expiratory pressure of 3 cm H_2_O (lowest value on the ventilator), which was adjusted according to the first blood gas analysis results. The ventilator setting was not changed after osmotherapy. Mean arterial blood pressure (MAP), heart rate, and rectal temperature were recorded throughout the study. Rectal temperature was maintained at 38.5–39.5 °C using a heating plate and a thermoisolation blanket. Balanced anesthesia was maintained using isoflurane (0.6–1 vol%, Forane, AbbVie Inc., Chicago, IL, USA) in a mixture of 1 l/min oxygen and 1.2 l air with an inspiratory oxygen fraction (FiO_2_) of 50–55 %, continuous intravenous infusion of fentanyl (0.4 μg/kg/min, Fentanyl Torrex, Chiesi Pharmaceuticals GmbH, Vienna, Austria), and the muscle relaxant pipecuronium bromide (0.6 mg/kg/h, Arduan, Gedeon Richter Plc., Budapest, Hungary).

Each animal was subsequently rotated into the prone position, and the right temporo-parieto-occipital area of the head was shaved. The skin and periosteum of the skull were incised and reflected, and bleeding was stopped by bipolar electrocoagulation. The margins of the exposed area were determined by the midline, the base of the right ear, the external occipital protuberance, and the right caudal supraorbital process. A 3-mm hole was drilled through the exposed skull and was increased in size using a mosquito pean. The final size of the cranial window was obtained using a Kerrisson rongeur. Bleeding from the diploe was stopped using bone wax. The dura mater was cut carefully around the edges of the cranial window using microscissors to minimize brain surface injury. The dimensions of the cranial window were approximately 12 × 8 mm, with intact arachnoid mater at the base of the window. A 15-min stabilization period was maintained after controlling bleeding. During this period, the wound was flushed frequently with sterile 37 °C normal saline, hemodynamic data were recorded, a sample of arterial blood was sent for laboratory examination (levels of blood gases, sodium, potassium, chlorides, and hemoglobin), a SDF probe was attached to the brain surface, and initial SDF imaging was performed.

### SDF imaging procedure

The SDF imaging probe was covered with a sterile plastic sheath and placed above the target tissue; a conventional hand-held technique was used. The sites of interest on the brain surface were selected randomly. Exposed tissues, other than those covered by the SDF imaging probe, were moisturized intermittently using 37 °C sterile normal saline. SDF imaging data were recorded digitally from three different areas (fields) within the site of interest for each animal at each measurement, and video clips lasting at least 20 s were recorded from each area (total of three video clips). Analysis of flow in larger vessels was used as a quality control measure to ensure that excessive pressure was not applied to the tissue [27].

### Experimental groups

Two animals were used to test the feasibility of this model, and 18 animals were included in the study. Two animals died during the instrumentation phase. Sixteen animals were randomized (a computer-generated random list of animals was used) to receive 3.75 ml/kg body weight of either 3.2 % HTS (HTS group) or 20 % mannitol (MTL group) solution administered intravenously over 15 min using an infusion pump after the initial SDF measurement. Both solutions had the same osmolarity (1099 mOsm/l) and were infused via a peripheral venous catheter. The 3.2 % HTS solution was prepared by the hospital pharmacy. The volume infused was equivalent to a dose of 0.75 g/kg body weight MTL. A second set of SDF imaging data were obtained, hemodynamic data were recorded, and a sample of arterial blood was sent for laboratory examination (including blood lactate levels) 20 min after infusing MTL or HTS. The animals were sacrificed at the end of the experiment using an overdose of thiopentone (30 mg/kg body weight).

The team members were blinded to the assigned groups while preparing the animals, acquiring data, analyzing the video clips, and conducting the statistical analysis. The administered solution was drawn up off-site and administered by an unblinded co-worker (a laboratory staff member).

### Off-line analysis

Video clips were randomly coded and analyzed offline by a single observer blinded to file order. Two clearest and most stable parts of each video clip (sequences) that met the software’s stability criteria were selected for the analysis. Flow in larger vessels was checked to ensure that excessive pressure was not applied during recording. A total of six sequences were analyzed per animal per measurement, and the average was used for subsequent calculations. The final on-screen magnification of the images obtained using the SDF imaging device was 325-fold the original, and the actual size of the field evaluated was 1280 × 960 μm.

Microcirculatory parameters were measured using AVA V3.0 software (AMC, University of Amsterdam, Netherlands). To decrease possible inter-observer variability, all analyses were performed by a single, blinded researcher (VDjr).

The following parameters were analyzed offline:

1. Total small-vessel density (SVD) and all-vessel density (TVD) were defined as the total length of the respective vessels inside the image divided by the total area of the image. Small vessels were defined as those with diameters ≤ 25 μm [27].
2. The DeBacker score, given in mm^−1^, was defined as the number of vessels crossing three arbitrary horizontal and three vertical equidistant lines (drawn on the screen) divided by the total length of the lines [27].
3. Microvascular flow index was calculated as an average value of the semiquantitative score (0 = absent flow, 1 = intermittent flow, 2 = continuous sluggish [slow] flow, 3 = continuous [normal] flow, 4 = hyperdynamic [fast] flow) of the microvascular flow in the four image quadrants, as assessed subjectively by an observer [27]. Absent flow was defined as no flow throughout the recorded image, intermittent flow as at least 50 % of the recorded time with no flow, sluggish flow as continuous but slow flow, and continuous flow as fast flow lasting throughout the recording.
4. The proportion of perfused vessels (PPV) was defined as the percentage of all visible vessels with at least sluggish flow. Perfused small vessel density (PSVD) and perfused vessel density (PVD) were obtained as SVD and TVD multiplied by the respective proportion of perfused vessels.

### Statistical analysis

A power analysis using an α error of 0.05 and β error of 0.2 was performed based on previously published brain microcirculatory data for rabbits [23] using MedCalc 7.6.0. (MedCalc Software, Ostend, Belgium). The sample size needed for the *t*-test (independent groups) to detect a 15 % difference in SVD or TVD and a 20 % difference in the DeBacker score was calculated. This calculation yielded a sample size of 16 subjects (eight subjects per group). The sample size was increased to 18 animals to compensate for potential dropouts and inaccurate predictions used for the power analysis.

The continuous variable results are presented as means ± standard deviations or as medians with interquartile ranges based on the results of a test of the normality of the distribution using a one-sample Kolmogorov-Smirnov test. Differences in sex were analyzed using Fisher’s exact test. The Mann–Whitney *U*-test was used to compare results between groups when the sample distribution was not normal (pH, PaCO_2_, PPV, MFI, dose of catecholamines, and lactate levels). An unpaired *t*-test was used to compare all other results between groups. A P value < 0.05 was considered to indicate significance. The statistical analysis was performed using MedCalc 7.6.0.

## Results

Sixteen animals completed the study. No differences were observed in the demographic variables or initial hemodynamic and laboratory data with the exception of heart rate, which was higher in the MTL group than that in the HTS group (Table 1).

**Table 1:** Characteristics of the experimental groups

	Group HTS (n = 8)	Group MTL (n = 8)	P
Weight (kg)	2.382 ± 0.227	2.363 ± 0.159	0.8415
Sex (male:female)	3:5	5:3	0.6193
Mean arterial pressure (mmHg)	66.3 ± 4.9	65.2 ± 5.9	0.6951
Heart rate (beats/min)	189 ± 14	204 ± 7	0.0230
Temperature (°C)	39.2 ± 0.6	38.9 ± 0.6	0.3837
pH	7.36 ± 0.05	7.35 ± 0.10	0.7662
PaCO_2_ (kPa)	6.91 ± 0.89	7.21 ± 2.62	0.7655
PaO_2_ (kPa)	21.2 ± 8.2	21.0 ± 8.9	0.9613
HCO^3^- (mmol/l)	29.4 ± 2.2	29.0 ± 4.4	0.8158
Hemoglobin (g/l)	105 ± 4	105 ± 8	0.9076

Weight, sex, mean arterial pressure, heart rate, temperature, blood gases, and hemoglobin values after initiating mechanical ventilation. HTS, hypertonic saline; MTL, mannitol

Table 2 lists the microcirculatory parameters. No significant differences were observed between the groups before the use of osmotherapy. After osmotherapy, lower values of PPV (P = 0.0379), PSVD (P = 0.0474), PVD (P = 0.0457), and MFI (P = 0.0207) were observed in the MTL group compared with the HTS group.

**Table 2:** Microcirculatory parameters

	Before osmotherapy			After osmotherapy		
	Group HTS (n = 8)	Group MTL (n = 8)	P	Group HTS (n = 8)	Group MTL (n = 8)	P
SVD (mm.mm^−2^)	7.44 ± 3.01	6.55 ± 2.36	0.5216	6.82 ± 1.95	5.86 ± 1.34	0.2699
TVD (mm.mm^−2^)	10.60 ± 2.44	10.06 ± 2.41	0.6636	9.99 ± 2.45	8.04 ± 2.00	0.1022
PSVD (mm.mm^−2^)	7.33 ± 2.95	6.43 ± 2.18	0.4978	6.70 ± 2.06	4.51 ± 2.00	0.0474
PVD (mm.mm^−2^)	10.37 ± 2.52	9.88 ± 2.45	0.7021	9.75 ± 2.60	6.73 ± 2.89^*^	0.0457
PPV (%)	97.1 ± 3.9	98.1 ± 3.1	0.5900	96.9 ± 4.7	79.8 ± 22.5^*^	0.0541
MFI	3.00 (2.97;3.00)	2.95 (2.79;3.00)	0.3282	3.00 (2.98;3.01)	2.66 (2.52;2.88)	0.0207
DeBacker score (mm^−1^)	6.59 ± 1.33	6.43 ± 1.57	0.8247	6.49 ± 1.58	5.32 ± 1.14	0.1128

*SVD* small vessel density, *TVD* total vessel density, *PSVD* perfused small vessel density, *PVD* perfused vessel density, *PPV* proportion of perfused vessels, *MFI* microvascular flow index, *HTS* hypertonic saline, *MTL* mannitol

^*^P < 0.05 vs Before osmotherapy

Table 3 compares the hemodynamic data and laboratory values, as well as the use of fluids and catecholamines, before and after osmotherapy. After osmotherapy, higher blood sodium levels were observed in the HTS group compared to pre-osmotherapy levels (P = 0.0007) and compared to MTL group (P = 0.0001).

**Table 3:** Hemodynamic and laboratory data and use of fluids and catecholamines

	Before osmotherapy			After osmotherapy		
	Group HTS (n = 8)	Group MTL (n = 8)	P	Group HTS (n = 8)	Group MTL (n = 8)	P
MAP (mmHg)	56 ± 7	56 ± 4	0.9449	55 ± 5	54 ± 7	0.7726
Heart rate (beats/min)	206 ± 13	213 ± 9	0.2439	210 ± 16	215 ± 7	0.4281
pH	7.34 ± 0.15	7.35 ± 0.19	0.8466	7.34 ± 0.1	7.33 ± 0.2	0.9044
PaCO_2_ (kPa)	6.86 ± 2.64	6.39 ± 2.71	0.7408	7.57 ± 2.47	7.25 ± 4.58	0.8644
PaO_2_ (kPa)	24.3 ± 9.5	22.5 ± 10.0	0.7151	23.3 ± 9.4	23.0 ± 9.7	0.9503
HCO^3^- (mmol/l)	26.6 ± 5.2	25.7 ± 3.8	0.6918	30.3 ± 4.2	26.9 ± 8.0	0.3139
Hemoglobin (g/l)	94.5 ± 6.3	91.9 ± 10.3	0.5523	97.1 ± 5.2	94.5 ± 21.1	0.7383
Sodium (mmol/l)	139.0 ± 0.9	138.6 ± 1.2	0.4365	142.2 ± 2.0^*^	137.5 ± 7.7	0.0001
Potassium (mmol/l)	3.2 ± 0.4	3.6 ± 0.7	0.1353	3.6 ± 0.5	3.8 ± 0.5	0.6041
Chlorides (mmol/l)	104.9 ± 1.9	104.0 ± 4.2	0.5845	107.0 ± 2.2	103.5 ± 5.5	0.1154
Glucose (mmol/l)	12.4 ± 2.3	13.8 ± 3.6	0.3811	13.1 ± 4.4	13.7 ± 7.1	0.8583
Lactate (mmol/l)	--	--		2.96 ± 3.97	3.00 ± 1.70	0.9808
Infused fluids (ml)	--	--		42 ± 9	44 ± 10	0.6266
Number of animals recieving catechalamines	2	4	0.6038	2	4	0.6038
Highest dose of catechoalmines (μg/h)	0.00 (0.00;0.30)	0.25 (0.00;3.00)	0.4418	0.00 (0.00;0.30)	0.25 (0.00;3.00)	0.4418

*MAP* mean arterial pressure, *HTS* hypertonic saline, *MTL* mannitol

^*^P < 0.05 vs Before osmotherapy

We observed a non-significant trend toward higher lactate levels (1.65 [1.20, 2.25] vs. 2.70 [2.00, 3.60], P = 0.1049) and lower arterial PaCO_2_ levels (6.43 [5.76, 9.42] vs. 5.2 [4.76, 8.54], P = 0.1605) in the MTL group.

**Fig. 1:**
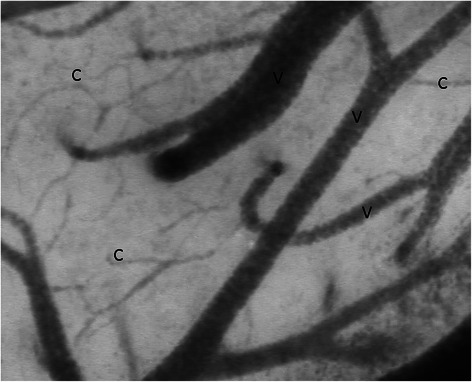
Representative sidestream dark-field image of rabbit cerebral microcirculation. Objective microscope (5×), on-screen 325×, size of image = 1280 × 960 μm. C, capillary; V, venule

Figure 1 provides a representative image of rabbit cerebral microcirculation.

**Fig. 2:**
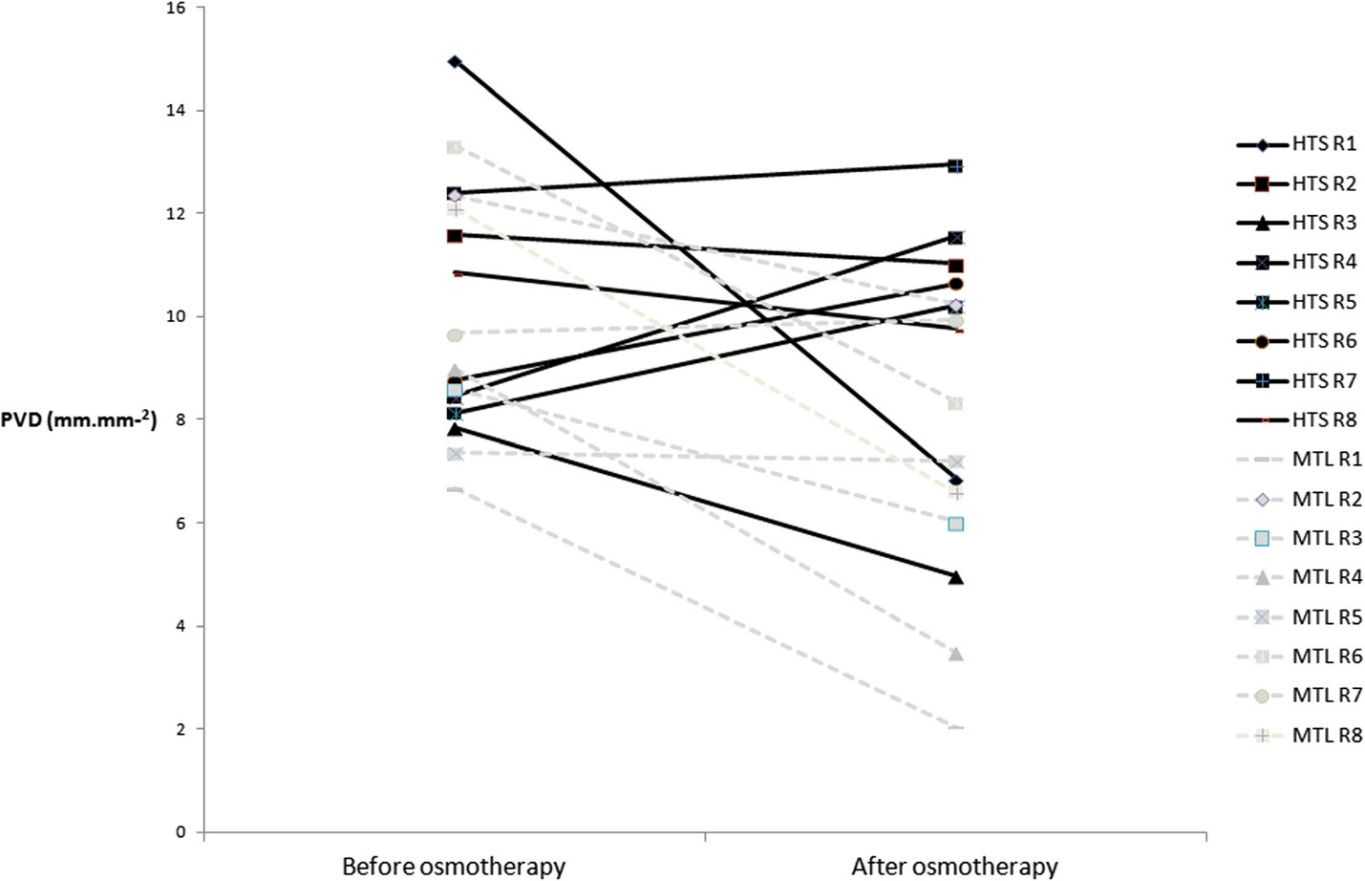
Individual pre- and post-treatment perfused vessel density values. HTS, hypertonic saline; MTL, mannitol; R1, R2, etc., number of rabbits per group

Figure 2 shows the pre- and post-treatment PVD values for each rabbit in both groups.

## Discussion

We demonstrated improved preservation of cerebral cortex microcirculation with HTS compared with MTL in an animal craniotomy model; the observed differences were caused mainly by a higher proportion of perfused vessels in the HTS group.

The diuretic effect is an important component of the MTL effect on the brain [5, 6, 28]. The diuretic response to MTL, which onsets almost immediately, is strongest during the first 10 minutes after MTL infusion [29]. MTL infusion has also been shown to decrease cardiac stroke volume after short-term improvement [29]. The strong diuretic effect of MTL may cause undesired hypovolemia. Rozet at al. described a significant rise in serum lactate levels during surgery in patients receiving MTL for brain debulking [4]. In contrast, HTS is considered a volume expander that improves regional blood flow [8], and it has a weaker diuretic effect [4, 5, 28]. Although we observed no group differences in lactate levels or other macrocirculatory parameters, the lower proportion of perfused vessel density could possibly be caused by reduced brain perfusion due to diuresis-induced or worsening hypovolemia, the severity of which was not associated with macrocirculatory compromise. Unfortunately, we did not measure urine or cardiac output or cerebral blood flow for technical reasons.

HTS is also considered more effective for brain relaxation and intracranial pressure control in patients undergoing neurosurgical procedures [6]. These effects may also account for the observed differences in microcirculation.

Hypertonic solutions may also modify blood viscosity, the interaction between endothelial cells and polymorphonuclear cells, blood rheology, and permeability of the BBB [11, 30]. A recent study reported no difference in BBB permeability after 15-min infusion of MTL or HTS [30]. In that study, the water content of brain tissue samples in both the MTL and HTS groups was significantly greater compared with controls, but there was no difference between the two experimental groups [30]. In contrast, another recent animal study [28], which used a 45-min HTS infusion time, reported higher brain water content in the HTS group. In theory, tissue edema could be associated with a lower number of visible vessels, e.g., smaller TVD and SVD values. Although we observed a trend toward lower TVD and SVD in the MTL group, the difference was not significant.

Changes in cortical brain microcirculation due to MTL or HTS have not been evaluated using SDF imaging. Sepsis-induced changes in brain microcirculation have been described using the same technology [25, 26]. Lower PVD values were associated with lower brain oxygen tension values. Intravital video microscopy of pial microcirculation has been used to observe the effects of MTL and HTS on the interaction between polymorphonuclear and endothelial cells in a model of trauma-induced brain inflammation [30], but changes in cortical perfusion have not been evaluated. Orthogonal polarization spectral imaging of the brain cortex during aneurysm surgery has been used to observe the small cortical blood vessels directly and to quantify their responses to hypocapnia, but the effects of osmotherapy have not been evaluated [31].

Our study had several limitations. The number of animals in each group was small, and higher numbers may have resulted in greater differences between the groups and decreased risk of false-positive results. An experimental group that underwent cannulation, craniotomy, and no osmotherapy would have provided a positive control and additional information about baseline injury, edema, and the osmotic agent responsible for the observed differences in microcirculation. The surgery or anesthesia may have played a role in the observed changes, although we tried to avoid local brain injury, minimize the number of manipulations, and avoid tissue desiccation by administering warm saline solution. We also performed a large craniectomy that prevented any potential influence from intracranial pressure; therefore, it may not be appropriate to generalize our findings to subjects with increased intracranial pressure. We visualized only pial vessels and the frontal cortex, and these areas may not be representative of deeper brain structures. We also used a rabbit brain, which has unknown similarities in blood flow regulation to those of the human brain., Therefore, it is not clear whether our results could be safely extrapolated to humans. Due to the positioning of the animals, which was unchanged during the study, we were unable to investigate microcirculation in other regions of potential interest (sublingual region). Differences in the time-to-peak effect and duration of action between MTL and HTS are also possible. A variable time-to-peak effect and duration of action have been described for both MTL and HTS [32], although some evidence suggests a possible shorter time-to-peak effect and lower rebound phenomena in subjects with brain lesions treated with HTS [33]. Changes in microcirculation after osmotic therapy were not measured at multiple timepoints, such that we cannot exclude the possibility that the observed effects may have been different if other timepoints had been chosen.

## Conclusions

Within the limitations of this study, our findings suggest that an equivolemic, equiosmolar solution of HTS preserves perfusion of the cortical brain microcirculation better than does MTL in a rabbit craniotomy model. A human study is warranted to ascertain whether similar effects occur in patients undergoing scheduled craniotomy for brain tumor surgery.
